# In silico assessment of the conduction mechanism of the Ryanodine Receptor 1 reveals previously unknown exit pathways

**DOI:** 10.1038/s41598-018-25061-z

**Published:** 2018-05-02

**Authors:** Leonard P. Heinz, Wojciech Kopec, Bert L. de Groot, Rainer H. A. Fink

**Affiliations:** 1 Medical Biophysics Unit, Medical Faculty, Institute of Physiology and Pathophysiology, Heidelberg University, 69120 Heidelberg, Germany; 2 Computational Biomolecular Dynamics Group, Max Planck Institute for Biophysical Chemistry, 37077 Göttingen, Germany; 3 Present Address: Department of Theoretical and Computational Biophysics, Max Planck Institute for Biophysical Chemistry, 37077 Göttingen, Germany

## Abstract

The ryanodine receptor 1 is a large calcium ion channel found in mammalian skeletal muscle. The ion channel gained a lot of attention recently, after multiple independent authors published near-atomic cryo electron microscopy data. Taking advantage of the unprecedented quality of structural data, we performed molecular dynamics simulations on the entire ion channel as well as on a reduced model. We calculated potentials of mean force for Ba^2+^, Ca^2+^, Mg^2+^, K^+^, Na^+^ and Cl^−^ ions using umbrella sampling to identify the key residues involved in ion permeation. We found two main binding sites for the cations, whereas the channel is strongly repulsive for chloride ions. Furthermore, the data is consistent with the model that the receptor achieves its ion selectivity by over-affinity for divalent cations in a calcium-block-like fashion. We reproduced the experimental conductance for potassium ions in permeation simulations with applied voltage. The analysis of the permeation paths shows that ions exit the pore via multiple pathways, which we suggest to be related to the experimental observation of different subconducting states.

## Introduction

Ryanodine receptors (RyRs) are large ion channels that play a pivotal role in the regulation of the free calcium concentration in living cells^1^. Their main purpose is gating calcium releases from internal storages, such as the sarcoplasmic and endoplasmic reticula^2–5^. RyRs are tetramers consisting of four identical protomers. The full receptor has a molecular mass of 2.2 megadaltons and its just over 5000 residues per protomer make it the largest known ion channel^6^. Three mammalian isoforms of RyR are known (RyR1 to RyR3): RyR1 is abundant in skeletal muscle, where it is responsible for the rapid release of calcium ions from the sarcoplasmic reticulum (SR)^6–8^. Subsequent to their release into the cytoplasm, the calcium ions bind to troponin-C and trigger muscular contraction^9,10^. Hence, RyR1 plays a crucial role in the excitation-contraction coupling of skeletal muscle (e.g.^11–13^). Mutations in the RyR1 isoform have been linked to malignant hyperthermia and central core disease^2,3^. RyR2 is responsible for calcium induced calcium release in cardiac muscle^14,15^, whereas RyR3 is found in smooth muscle and brain tissue^16^.

Ryanodine receptors gained a lot of attention recently^17,18^, after multiple authors published cryo electron microscopy (cryo-EM) data of the receptor in unprecedented resolution, reaching down to 3.8 Å in the closed^19–23^ and 4.1 Å in the open conformation^21–24^. Although Molecular Dynamics (MD) simulations on homology models of RyR1 have been carried out before^25–28^, the availability of these high resolution structural data sets allows for direct simulation of RyR1.

In this manuscript, we assessed the conduction and selectivity mechanisms of RyR1 by using MD simulations. Therefore, we first constructed a full model of RyR1, based on the most recent cryo-EM data. To address the conduction mechanism of RyR1 and its energetics, we calculated potentials of mean force (PMFs) along the central pore for different ion types and identified key residues, directly responsible for the conduction of ions in RyR1. Furthermore, we investigated the conduction pathways by performing ion permeation simulations, in which ions are driven across the channel by an externally applied electric field, closely mimicking electrophysiological measurements, and found new ion exit pathways in the channel.

## Methods

### Molecular dynamics simulations

All MD simulations were carried out using the software package Gromacs 5.1^29–33^ and the CHARMM36 force field^34–37^. The system was propagated in time using the leap frog integrator with a time step of 2 fs, with the only exception being the first equilibration run, in which a reduced time step of 1 fs was used for stability reasons. During the simulations, H-bonds were restrained using the LINCS algorithm^38^, long range electrostatic interactions were treated using the Particle-Mesh Ewald (PME) method^39^ and van-der-Waals and Coulomb cutoffs were set to 1.2 nm. The TIP3P water model^40^ was used and all molecular visualizations were done with the VMD software^41^.

### Receptor modelling

Although cryo-EM datasets of the open-state receptor are available, the quality of the closed state data is significantly better compared to the open state. Therefore, we first constructed an atomistic model of the closed receptor, which was equilibrated and later transitioned into the conductive state by adding biasing forces to the simulation in a steered MD-like fashion.

**Figure 1:**
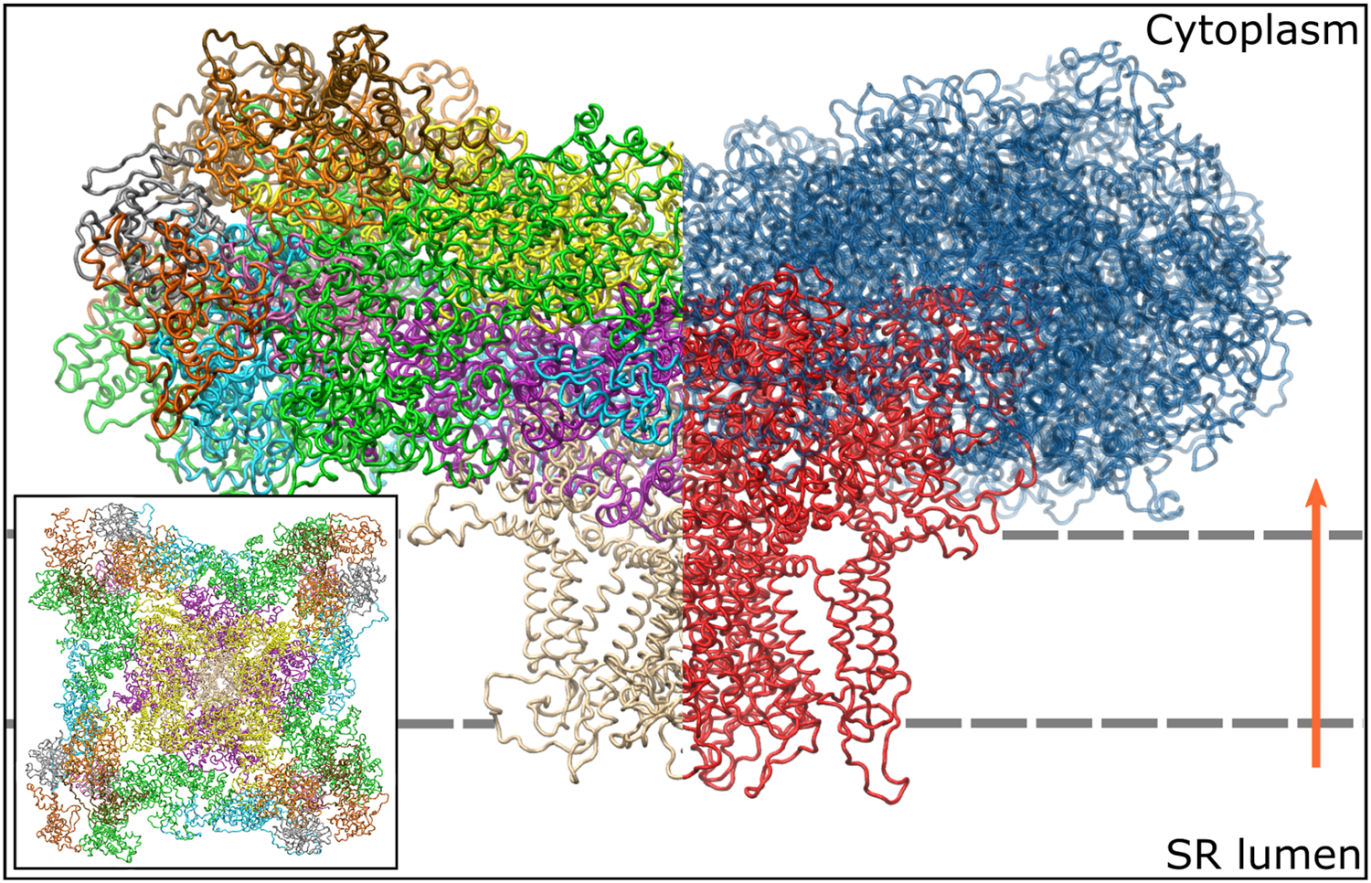
Side view of the RyR1-model. The left side is color-coded according to the domains of the receptor. Major domains are the N-terminal domain (), the handle domains (), the helical domains (), the central domain () and the channel domain (). The right side highlights the reduced model, consisting of the central and channel domains, in red. The orange arrow indicates the direction of the calcium current from the SR into the cytoplasm. A top-view is shown in the inset.

A full structural model of the closed RyR1 was constructed utilizing data obtained by Yan *et al*.^19^, Rouslan *et al*.^21^ and Zalk *et al*.^20^ (see Fig. 1). Loops, unresolved in all of the referenced data sets, were modeled using the Modeller software with the DOPE scorer^42–45^. In addition to loops, the structures contain five large unresolved segments, which are partially disordered^19,20^. For two of those, located in the SPRY3 and between the handle and central domains, Modeller was used to construct homology models separately, which were later added to the model. The further unresolved parts are contained in the handle domain, between the central and channel domains as well as in the channel domain and are likely disordered^19,20^. In the first and last case, predicted models were used for the simulations^46^. The second disordered part, almost 300 residues in size, needed to be excluded from the model. Therefore, the residues A4269 and L4544 were directly connected.

The obtained model of RyR1 was relaxed using Gromacs in a multistep fashion. First, the system was subject to 20000 steps of *in vacuo* energy minimization. After that, the model was solvated using approximately a million water molecules and charge neutrality was achieved by adding 360 Ca^2+^ ions, to create a realistic environment for the luminal pore. Further energy minimization was conducted on the full system, containing the protein, solvent, and ions, until the maximum force (on any atom) consistently reached values below 1000 kJ/mol/nm. For additional relaxation, the solvated receptor was simulated for 100 ps, while the temperature was kept constant at 300 K, using a velocity rescaling thermostat^47^. During this simulation, harmonic restraints were placed on all protein atoms. To allow relaxation of the side chains, an additional simulation run, lasting 200 ps, during which only Cα atoms were restrained, was conducted. The remaining restraints were reduced in a stepwise fashion to every 4th Cα atom and later to every 16th Cα atom in simulation runs of 100 ps length. To stabilize the well resolved pore (consisting of the central and channel domains), harmonic restraints were imposed on every 16th Cα atom of the pore during an additional relaxation run, lasting 2.5 ns. In total, the system could relax for 3 ns as a NVT ensemble. To obtain a relaxed system at atmospheric pressure, further 7 ns were simulated using a Berendsen barostat^48^ (approximate NPT ensemble).

### Membrane embedding

A small POPC patch, which had been equilibrated for 35 ns using the CHARMM36 force field^49^, was used to form a suitable membrane. It was enlarged to match the size of the receptor by concatenating multiple copies of it and the enlarged patch was subject to further 2 ns of NPT equilibration. The protein was embedded into the membrane, using the program g_membed^50,51^. With this algorithm, the receptor was scaled to 0.1 of its size along the dimensions parallel to the lipid bilayer. Lipids, intersecting with the shrunken protein, were removed and the receptor was gradually scaled back to its original size within 2000 molecular dynamics steps. After embedding, the complete system was subject to additional relaxation, lasting 1 ns (NVT) and 1.5 ns (NPT).

### Adding ions

The calcium ions, used to neutralize the system during equilibration, were removed. New ions were added to the compartments of either side of the membrane. In particular, 3.2 mmol/L of Mg^2+^ and 10 mmol/L of Ca^2+^ ions were added to the luminal side of the system. On the cytosolic side, 140 mmol/L of K^+^ were added and the entire system was neutralized using Cl^−^ ions. Whereas a physiological amount of potassium ions was used, the luminal calcium concentration is typically reported to be 1 mmol/L^2^. Due to the small system sizes accessible by MD simulation, divalent ions were added at the very high end of experimentally observed concentrations. In total, 37 Ca^2+^, 12 Mg^2+^, 1274 K^+^ and 652 Cl^−^ ions were included. To maintain the asymmetric ion concentrations in the single-membrane system during simulation runs under periodic boundary conditions, an energy step method similar to the one developed by Khalili-Araghi *et al*.^52^ was implemented. Here, spatial restraints were imposed upon all cations using a step-shaped potential. They were kept in their respective compartment by a repulsive force, that acted on the ions within a narrow transition region around the periodic boundary. A force of 10000 kJ/mol/nm turned out to allow for stable simulation runs, while simultaneously being strong enough to reliably restrain all ions in their compartments. Further details on the usage and implementation of the energy step method are available in the supplementary material.

### Equilibrating and obtaining a reduced system

The entire simulated system was contained in a box of 36.6 × 36.6 × 21.5 nm^3^ in size and consisted of three million particles. The full system was equilibrated for 100 ns, using a Parrinello-Rahman barostat^53–55^ for semiisotropic pressure coupling. Although fully equilibrating the entire receptor in 100 ns was impossible, the simulation run was sufficient to converge the root-mean-square deviation (RMSD) of the relevant subsystem, which would later make up the reduced model (see Supplementary Material Fig. 2).

For all further simulation runs, the equilibrated model was reduced to the central and channel domains, which form the pore of the receptor. Due to the large size of RyR1, a reduced model is more suitable to study potentials of mean force along the central axis and the ion conduction mechanisms. Therefore, all residues up to A3680 were deleted. Because of the mushroom-like shape of the receptor (see Fig. 1), this procedure allowed for a dramatic reduction of the membrane patch size and as a consequence, a drastically smaller system and thus a reduced computational load.

The reduced system was resolvated and ions were added again, as described above. To avoid conformational changes due to the missing parts of the receptor, harmonic restraints were placed upon the interface of the reduced model to its removed parts. For this purpose, a force constant of 1000 kJ/mol/nm^2^ was used for all C*α* atoms of residues A3680 to L4170. The reduced system consisted of 700000 particles.

### Obtaining a model of open state RyR1

The model was steered into a conductive state using the recent structural data by des Georges *et al*.^22^ of open-state RyR1 (PDB: 5TAL). All C*α* atoms of the pore region (defined by V4820 to T4970), including the pore-forming helices (S1–S6) and the selectivity filter, were dragged into their new positions as dictated by the cryo-EM data within 5 ns. This was done using harmonic potentials with force constants of 500 kJ/mol/nm, applied through the flooding algorithm^56,57^. To equilibrate the structure of the open-state receptor, constraints were maintained on every forth C*α* atom for a simulation run of 30 ns. After restrained equilibration, we observed a converged Cα RMSD of 0.75 nm for the entire reduced system and 0.35 nm for the pore region, compared to the cryo-EM structure. The value decreased to 0.3 nm if the extremely flexible luminal loops were excluded from the calculation.

Further 30 ns were simulated without restraining the structure in its conductive conformation, to see if the channel would spontaneously close again. No significant closure movement was observed (see Supplementary Material Fig. 3). Additionally, longer unrestrained equilibration yielded an increase of RMS distances by only 0.1 nm compared to the restrained run, indicating that the open structure is stable within the accessible timescales (see Supplementary Material Fig. 4). Therefore, no open-state restraints were used during the 0.6 ns-short runs during umbrella sampling. However, to guarantee the channel remains fully open during the longer 100 ns-long ion permeation runs, harmonic open-state restraints were placed on every forth C*α* atom of the pore region during the applied voltage runs, as described above.

### Calculating potentials of mean force

Potentials of mean force (PMFs) were calculated for barium, calcium, magnesium, sodium, potassium and chloride ions across the central axis of the ryanodine receptor in its open state. Each PMF was computed using umbrella sampling and the weighted histogram analysis method (WHAM)^58–60^, based on 209 umbrella windows. Each window was prepared by exchanging the ion of interest with a water molecule. Although the typical distance between water molecules is larger, a window spacing of 0.7 Å was used. This was done by taking four snapshots from the last 80 ps of the proceeding equilibration run. For each umbrella window, the snapshot that allowed for optimal spacing was used. During the umbrella sampling simulations, the ion was held in place by a harmonic biasing potential with a spring constant of 500 kJ/mol/nm^2^ in axial direction. Each window was simulated for 0.6 ns without open-state restraints, of which the first 0.1 ns were excluded from analysis. Because the channel is typically occupied by many ions simultaneously, other ions were not restrained from entering the pore during the umbrella sampling. However, no further ions were present in the narrow hydrophobic core, ranging between −2 nm and 1 nm, during umbrella sampling. We calculated cyclic PMFs with g_wham^60^ and obtained error estimates by using 200 bootstraps.

**Figure 2:**
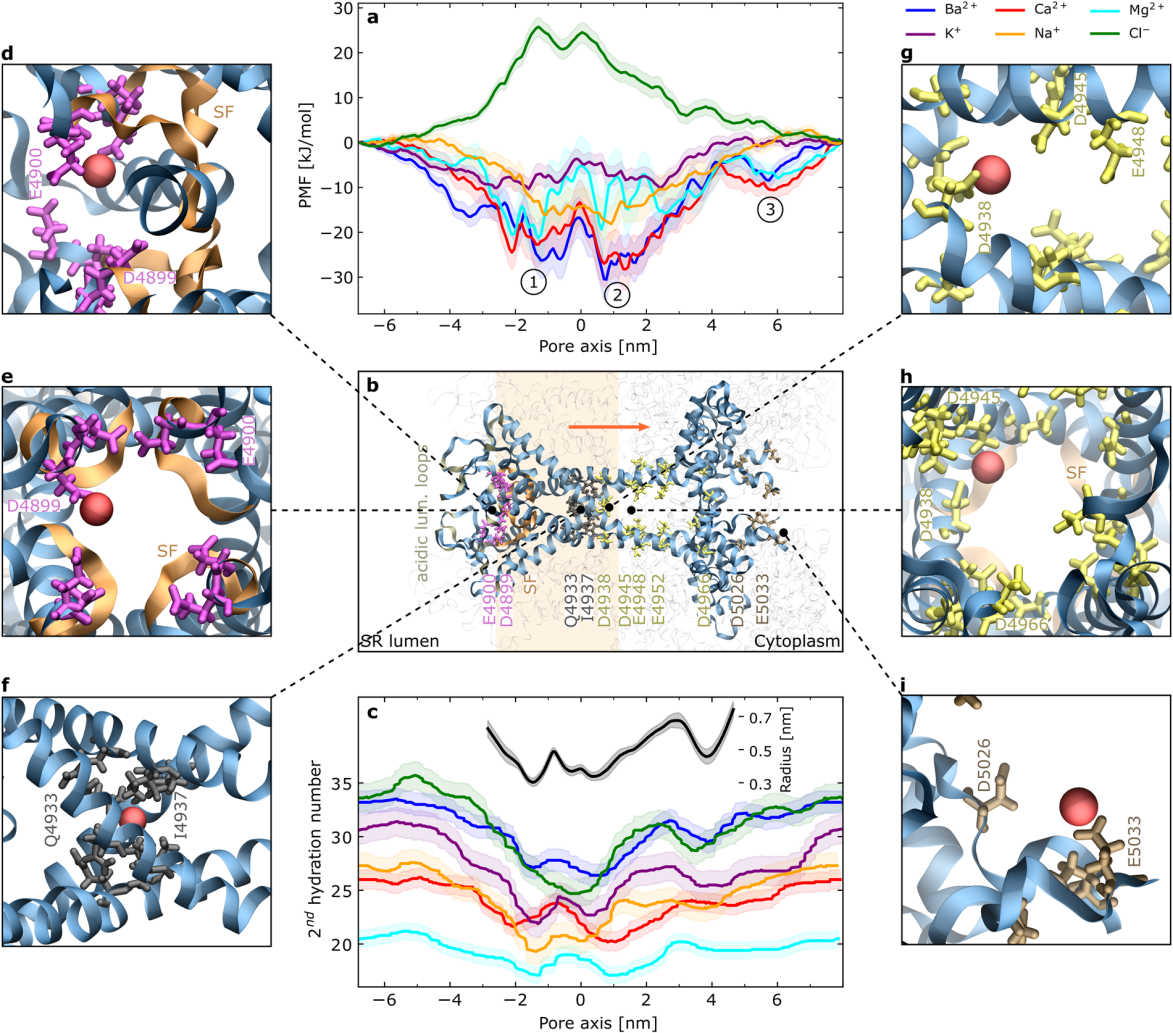
(**a**) PMFs across the open pore. Data shown for Ba^2+^ (), Ca^2+^ (), Mg^2+^ (), Na^+^ (), K^+^ () and Cl^−^ () ions. The main PMF basins are labeled as ①, ② and ③. (**b**) The pore of RyR1, aligned with the pore axis of the panel above. Key residues are highlighted in color, depending on which PMF feature they contribute to; the orange arrow indicates the direction of ion permeation. The semitransparent bar indicates the position of the membrane. Panel (**c**) shows the number of water molecules in the second hydration shell of each ion type across the pore. The inset shows the pore radius and its standard error, based on the structures of all umbrella windows, calculated using HOLE^101^. The panels (**d**) to (**i**) show a calcium ion interacting with key residues, as labeled in panel (**b**). Note that 4 copies of each residue are shown, since RyR1 is a tetramer.

From the PMF we obtained standard free energies of binding (Δ*G*^0^) using the method derived by Doudou *et al*.^61^ without restraints perpendicular to the reaction coordinate. The calculation was carried out as described by Pongprayoon *et al*.^62^ in the supporting information of the cited paper, who used the formalism in a similar context previously. Ions were considered in bound state between −6.0 nm and 7.5 nm along the pore axis and considered unbound (in bulk phase) outside the interval (see Fig. 2a). We estimated the effectively sampled area perpendicular to the reaction coordinate in analogy to equation (8) in^61^:

$$A={\int }_{-\infty }^{\infty }{e}^{-\frac{{x}_{1}^{2}}{2{\xi }_{1}^{2}}}d{x}_{1}\,{\int }_{-\infty }^{\infty }{e}^{-\frac{{x}_{2}^{2}}{2{\xi }_{2}^{2}}}d{x}_{2}=2\pi {\xi }_{1}{\xi }_{2},$$

where ${\xi }_{1}^{2}$ and ${\xi }_{2}^{2}$ are the eigenvalues of the covariance matrix of the bulk phase windows in the plane perpendicular to the reaction coordinate. The effectively sampled area amounted to 3.09, 3.04, 5.15, 9.09, and 3.30 nm^2^ for Ba^2+^, Ca^2+^, Mg^2+^, K^+^, and Na^+^ respectively.

To obtain a better handle on the electrostatic contributions to the PMF, we calculated the electrostatic potential across the channel by solving the Poisson equation. The calculation was done using the APBS program^63^, using dielectric constants of 2 and 78.5 for protein/membrane and solvent respectively.

### Conducting ion-permeation simulations

Our initial attempts to perform ion permeation simulations with an applied voltage using calcium ions were unsuccessful, as the ions tended to get stuck in the pore. Unfortunately, the very high affinity of RyR1 for calcium ions renders direct permeation simulations impossible. This is due to the well known problem of over-affinity for divalent ions in classical, additive force fields, which will be addressed further in the Discussion. Therefore, we decided to use monovalent potassium ions instead of divalent calcium ions for the ion permeation simulations in the conductive state of RyR1. All ions were removed from the system and new potassium ions were added in symmetric concentrations of 140 mmol/L to either side of the membrane. An external electric field was added by specifying it in the Gromacs parameter file. The strength of the electric field *E*, specified in units V/nm, was chosen such that the product *E* · *L* with the box dimension in axial direction (*L* = 14.626 nm) resulted in voltages of −0.2, 0.0, 0.2, 0.3 and 0.5 V^64^. Although high in physiological terms, voltages of this strength are commonly used in MD simulations and have proven to yield good results in the past^65–67^. Of each run, lasting 100 ns, the first 10 ns were excluded from analysis. Ionic currents were determined by counting the net number of ions that crossed the periodic boundary parallel to the membrane. Additionally, a further run was conducted using a voltage of 1.0 V, which lasted 30 ns. Again, the first 10 ns were excluded from analysis.

### Data availability

The datasets generated during and/or analysed during the current study are available from the corresponding author on reasonable request.

## Results

### Umbrella sampling

All potentials of mean force of the open-state receptor are plotted in Fig. 2a. Additionally, the maximum energetic wells or barriers as well as the standard free energies of binding for all cations are summarized in Table 1.

**Table 1:** Free energies for the different ion types.

Ion	Δ*G*_PMF_	Δ*G*^0^
	[kJ/mol]	
Ba^2+^	−30.9 ± 4.7	−30.9 ± 3.7
Ca^2+^	−28.3 ± 5.4	−30.1 ± 3.8
Mg^2+^	−21.5 ± 6.8	−23.6 ± 4.0
K^+^	−10.6 ± 2.4	−14.1 ± 1.5
Na^+^	−18.3 ± 4.2	−20.5 ± 3.3
Cl^−^	+25.9 ± 2.3	

The deepest PMF well (−) or highest barrier (+) for each ion type is given by Δ*G*_PMF_. Δ*G*^0^ is the derived standard free energy of binding.

Clearly, the ion affinity depends heavily on the ionic charge, which is due to the negative net charge of the receptor pore. Therefore, the receptor shows greater affinity to divalent cations than for monovalent cations and it is repulsive for the chloride anion. The PMFs for all cations show similar features: There are two main basins around −2 nm and +1 nm, respectively, of which the first one is split into two basins by a small peak in the profile for Ba^2+^, Ca^2+^ and Mg^2+^. The two main basins are separated by a free energy barrier at 0 nm in the center of the pore. Furthermore, the divalent ions show affinity for a minor third binding site, located at 6 nm in Fig. 2a.

To explain those features, we examined the umbrella windows at relevant points and highlighted as well as labeled key residues of the pore in Fig. 2b. Additionally, close-up renderings of a calcium ion interacting with some of the residues are shown in Fig. 2d–i.

Starting on the luminal side of the SR membrane, the pore creates an attractive environment for cations through the abundance of negatively charged residues in the flexible, acidic luminal loops. In particular, the presence of residues E4867, D4868, E4869, D4870, D4877, D4878 as well as E4902, D4903, D4907 and E4908 directly in front of the pore contribute to a local increase of cationic concentrations.

At the entrance of the pore around 2.5 nm, its radius decreases quickly towards the selectivity filter (SF), a GGGIGDE motif, as shown in Fig. 2c. Two rings of glutamate and aspartate residues (E4900 and D4899) mark the beginning of the filter and contribute to the first PMF basin ①. As shown in Fig. 2d and e, the ion makes contact with the negatively charged carboxylate groups of either of the four copies of the residues in the tetrameric protein.

Slightly further down in the SF, the pore reaches its constriction site of 3 Å at G4894. Further towards the cytoplasmic side and inside the hydrophobic transmembrane part, two rings, formed by Q4933 and I4937 residues, create a secondary constriction site. Comparison with the equilibrated closed structure shows that these residues act as a gate, as it was also seen by others^68^. In this case, I4937 rotates into the pore and blocks it completely. In the open state, side chains only slightly extend into the pore, as depicted in Fig. 2f. Therefore, the free energy barrier at around 0 nm is likely caused by a combination of a small steric barrier due to Q4933 and I4937 and the free energy penalty for the hydrated ion in the hydrophobic transmembrane section. This also suggests that the PMF minimum ① is co-formed by the restrictions by G4894 and the hydrophobic core to either side and the consequentially increased entropy of the ion inside the cavity of increased radius between the two constriction points.

The ring of isoleucine residues (I4937) is directly followed by a negatively charged ring of aspartate residues (D4938). Since the channel has a radius of only 3.5 Å at this point, all four acidic residues strongly interact with the permeating cation. As shown in Fig. 2g, the ion makes contact with at least one of the four copies of the residue, while the remaining three residues are still close enough to interact with the ion. However, it seems that D4938 is not single handedly responsible for the second main PMF basin. Instead, further acidic residues, such as D4945, E4948, E4952 and D4699, extend into the channel and contribute to basin ②, as the pore is getting wider. The ion makes contact with each of those rings, as shown in Fig. 2h exemplarily, while the preceding and succeeding rings are still close enough for electrostatic interaction.

PMF basin ③ is caused by E5033 and to a lesser extent by D5026. At this point, the E5033-residues of adjacent protomers are already more than 2 nm apart, so that the PMF is dominated by the protomer that the ion binds to, whereas the remaining three are too far away for strong interactions.

**Figure 3:**
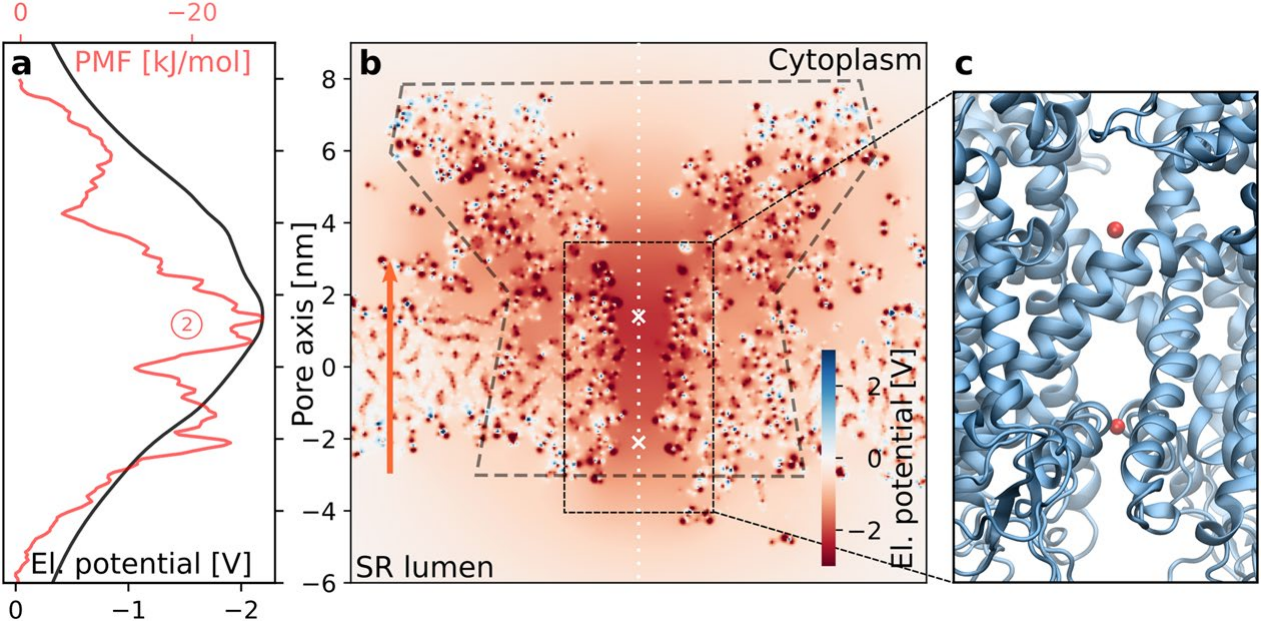
(**a**) One-dimensional electrostatic potential along the channel axis (white dotted line in panel (**b**)). The PMF for calcium is shown in red to aid orientation. (**b**) The two-dimensional electrostatic potential on a slice across RyR1. Dashed lines mark the outline of the channel to aid orientation. The locations of the two main PMF basis are marked by white crosses. The orange arrow indicates the direction of the calcium current. (**c**) Two red spheres were placed in the main PMF basins.

To check if the PMF basin ② is indeed not caused by a single amino acid, but rather by the electrostatics of many residues combined, we quantified the influence of the acidic residues on the electrostatic environment by numerically solving the Poisson equation for the open-state RyR1. Indeed, the results in Fig. 3 show a strong and attractive potential for cations. In particular, basin ② coincides with the minimum of the electrostatic potential across the channel axis.

Concerning the different ion types, Ba^2+^, Ca^2+^ and Mg^2+^ show the highest affinities. Barium and calcium show similar profiles: both with an approximate PMF depth of 30 kJ/mol. The PMF for magnesium is only 21.5 kJ/mol in depth. All three divalent ions bind similarly strongly to D4899 and E4900 in basin ①. Barium has a more pronounced basin ① compared to the other divalent ions, which is plausible as it is the largest of the three and thus the steric penalties due to the constriction sites to either side are larger. The Mg^2+^ ion does not bind as strongly to the second binding site as the other divalent cations, but all three show distinct dips in the profile at the positions of D4938, D4945, E4948, E4952, and D4966, while those dips are especially prominent for magnesium. The behavior of all three divalent ion types is almost identical at the third basin. The standard free energies of binding show an identical trend, as Ba^2+^ and Ca^2+^ bind strongly (−30.9 and −30.1 kJ/mol respectively) and Mg^2+^ follows with a standard free energy of −23.6 kJ/mol.

Monovalent cations like Na^+^ and K^+^ are also attracted by the receptor, although the affinity is considerably smaller compared to divalent cations. Potassium ions show the weakest PMF well of only 10.6 kJ/mol and the least attractive standard free energy of binding of −14.1 kJ/mol, which indicates that with no further cations present, RyR1 is a good conductor for potassium ions. Sodium ions have a more attractive standard binding free energy of −20.5 kJ/mol. Furthermore, Na^+^ ions seem to be almost unaffected by Q4933 and I4937, as the PMF of sodium does not show a free energy well in the center. The PMF for chloride reveals that RyR1 is strictly a cation channel.

To explain the differences in the PMFs of ions with identical valency, it is worth looking at the behavior of their hydration shells. We calculated the number of water molecules in the first and second hydration shell for all ion types across the pore, but only the profile of the second hydration number is shown in Fig. 2c. The first hydration number remained almost constant for all ions, as they kept more than 85% of their first hydration shell across the entire pore axis. With respect to the 2nd hydration number, we find that the profiles closely follow the pore radius. The ions are the most dehydrated at the constriction site caused by the SF, at the secondary constriction site as well as at D4938, where the ions bind closely to the residue and the pore is still narrow. Unsurprisingly, the size of the hydration shell as well as the amount of dehydration depend on the ionic size. In particular, large ions such as chloride, potassium and barium are dehydrated to a much greater extent than the small magnesium ion. Mg^2+^ is the smallest of all tested ions and as visible in Fig. 2c, it hardly loses water molecules from its second hydration shell. Therefore, the ion remains well shielded against the electrostatic influence of acidic residues, which could explain its small interaction free energy with the pore.

Although sodium ions are considerably smaller than potassium ions, Fig. 2c shows that Na^+^ is dehydrated to a similar extent as K^+^. Its smaller size could explain why sodium is almost unaffected by the steric and hydrophobic barrier of Q4933 and I4937. As previously seen with sodium channels^69^, its higher charge density yields stronger interactions with acidic residues, such as D4938, resulting in a larger binding affinity compared to potassium ions.

Based on the much larger affinity for divalent ions at basin ①, we expect divalent ions to substitute monovalent ions at this point and subsequently block them from permeating. We tested this hypothesis in permeation simulations with applied voltage.

### Ion permeation simulations

We have already shown that K^+^ ions yield a reduced standard binding free energy of only −14.1 kJ/mol, while at the same time showing a similarly shaped PMF compared to Ca^2+^ ions. We therefore expected to see a conduction behavior similar to calcium ions, in time scales that are currently accessible for simulations. Fortunately, RyR1 is well examined experimentally for both Ca^2+^ and K^+^, as many experiments were in fact done using potassium ions^70–73^. Hence, the comparison to experiments is possible and could shed light on the general ion permeation mechanism.

The ion permeation simulations allowed for a direct measurement of the current-voltage (IV) dependence of potassium ions through the receptor.

**Figure 4:**
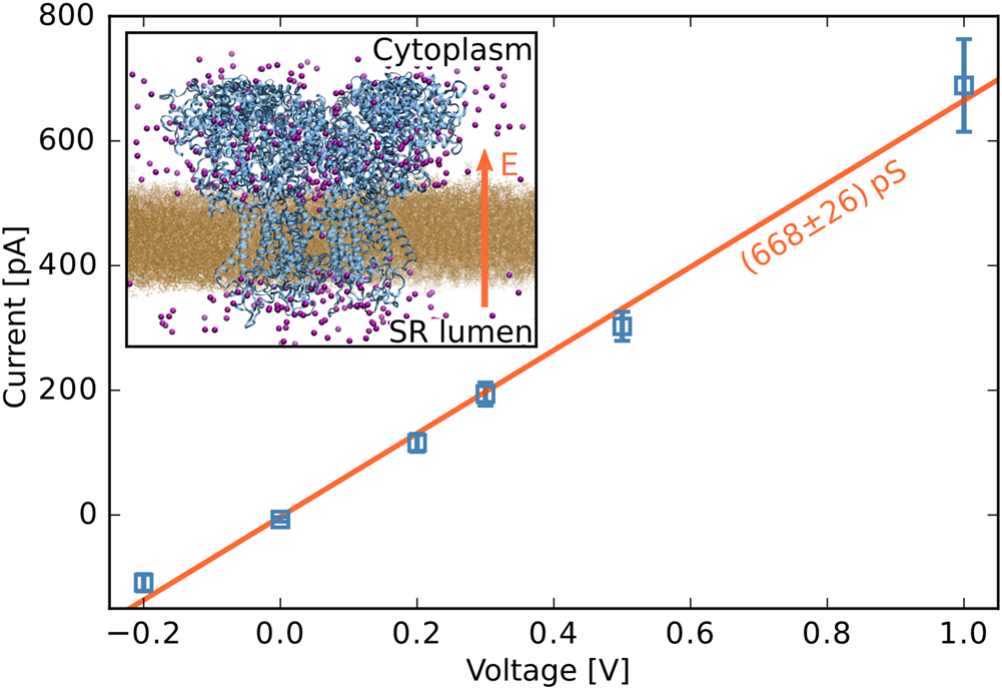
Current-voltage relationship as determined for potassium ions of the open-state RyR1. A linear fit to all data points from −0.2 to 1.0 V reveals a conductance of (668 ± 26) pS at 140 mmol/L. The inset shows a rendering of the simulated system; the arrow symbolizes the direction of the electric field added to the simulation and thus also indicates the direction of permeation.

Figure 4 shows a linear IV relationship over the entire tested voltage range, even for the unphysiological value of 1 V. A polynomial of first order was fitted to the data to determine conductances of (668 ± 26) pS at a symmetric physiological concentration of 140 mmol/L. The result is in good agreement with the experimental values between 700 and 800 pS at concentrations of 210^74^ to 250 mmol/L^71–73^ and is larger than reported values for calcium ions of roughly 100 pS^75^.

To determine the role of residues D4899 and E4900 in ion selectivity, we performed a second simulation run of 60 ns at 0.3 V, but saturated each of those eight acidic residues with a calcium ion at its carboxylate group. Indeed, the current decreased from (194 ± 19) pA in the calcium-free case to only (38 ± 11) pA with calcium present. The result indicates that the presence of calcium ions at these high-affinity binding sites imposes a rate limiting energetic barrier for potassium ions. The respective residues therefore serve as a filter for divalent ions over monovalent ions, which was also seen experimentally^71,72^.

**Figure 5:**
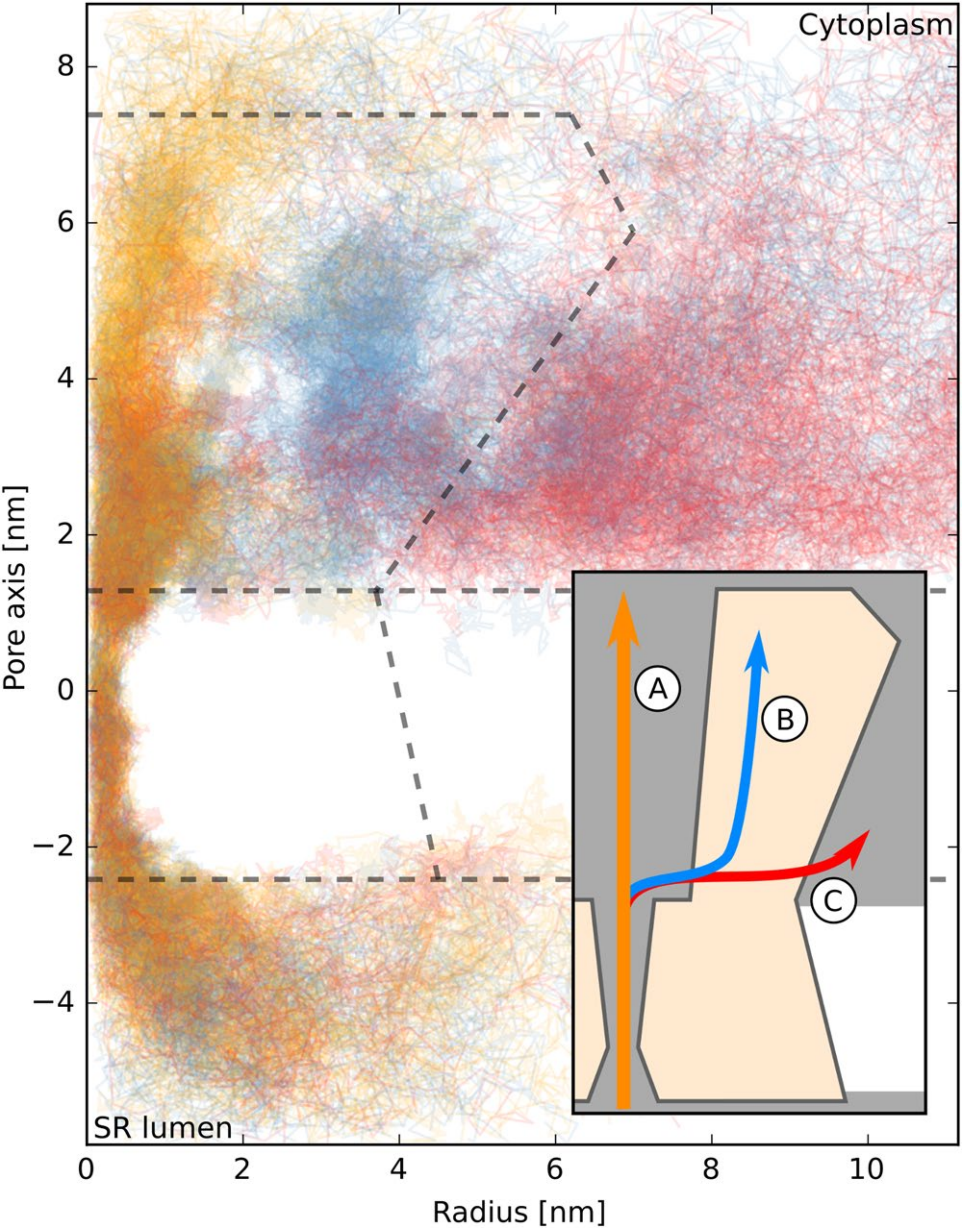
Ion trajectories of the simulation at 0.3 V are visualized by plotting axial coordinate against the distance to the pore axis (radius). Each trajectory is classified and color coded based on the path taken: Ⓐ , Ⓑ , Ⓒ . The outline of the membrane and the receptor are shown by dashed gray lines for orientation. The inset shows a schematic sketch of the 3 modes of exiting the receptor with their respective labels.

Furthermore, we analyzed the ion trajectories in more detail as depicted in Fig. 5. While all ions passed through the central transmembrane pore, the channel splits into multiple exits at the location of the second main PMF basin. Three modes of exiting the channel into the cytoplasm were observed. Most ions continued drifting in axial direction and exited trough the central pore (Ⓐ). Others moved into one of the water-filled clefts between the four protomers and exited either by diffusing in axial direction inside the cleft (Ⓑ) or by moving laterally (Ⓒ). In the latter case, ions left the receptor on the cytoplasmic side parallel to the membrane. Most, but not all of the ions that left the central pore sideways did so through a window, spanned by R4944, E4948, K4951, E4952 of one monomer and D4945, E4946, and E4949 of the monomer on the other side of the cleft. We propose these residues as targets for a direct experimental validation through mutagenesis studies.

To quantify this observation, the path of each pore-passing ion was analyzed and classified into one of the three pathways using a heuristic approach described in the supporting material.

A classified and color coded set of trajectories is shown in Fig. 5.

Taking the values of simulated external voltages up to 0.5 V into account, 53% of trajectories exited via pathway Ⓐ, whereas 25% and 17% exited through Ⓑ and Ⓒ, respectively. The remaining 5% of trajectories could not be classified uniquely according to the criteria described in the supporting material. We could not observe a clear correlation between the external voltage and the number of times a specific exit was chosen in the voltage range from −0.2 V to +0.5 V. However, in the additional simulation conducted at an external voltage of 1 V, 80% of trajectories exited via pathway Ⓐ, as the strong external electric field biases the ionic movement in axial direction.

## Discussion

We presented MD simulations on the RyR1 to shed light on the atomistic processes involved in ionic permeation. To address the conduction mechanism and its energetics, we performed umbrella sampling simulations for different ion types and obtained potentials of mean force and standard binding free energies. Major binding sites as well as the responsible key residues were identified. Furthermore, we conducted permeation simulations with an applied voltage and observed previously unknown conduction pathways, in which the ions exit the channel into the cytoplasm through the side of the receptor.

Our study is based on the latest cryo-EM data, which enabled us to perform atomistic simulations of ion conduction in RyR1 for the first time. The used cryo-EM structures, that represent the closed conformation of the receptor, have the highest resolution of all available data sets, especially as compared to these representing open conformations. Therefore, a model of closed RyR1 was built based on these structures and subsequently equilibrated, and later reduced to the significant domains and transitioned into the open state. With the final RMSD of 0.35 nm in the relevant pore region compared to the published open structure by des Georges *et al*.^22^, we believe we have built a realistic model of the receptor. Comparison of the radius profile of our model, as shown in Fig. 2c, with the profile published by des Georges *et al*.^22^, yields a qualitative agreement. Both profiles show a main constriction site of 3 Å, formed by G4894 in the SF, but we note that after equilibration, the sidechains of I4937 and D4966 extend further into the pore than in the cryo-EM structure. However, the small Cα RMSD of the pore region to the cryo-EM structure (0.35 nm and 0.3 nm without luminal loops) show that the most important part of the channel is well modeled and very stable. As indicated by the overall RMSD of 0.75 nm, larger deviations exist in the peripheral channel regions, where fewer residues were fully resolved by cryo-EM. Furthermore, the reduced model introduces a general caveat to our simulation results; however, the PMFs are typically dominated by local, short range interactions^76^. Also, the deleted part of the pore is already multiple nanometers in width and thus a direct influence on the conductance and the conduction mechanism seems unlikely.

Our simulations included a pure POPC membrane, which is commonly used for simulations and has been shown to be a good model of the SR membrane^77,78^. Although phosphatidylcholines (PC) lipids are found in the SR membrane in large quantities, pure POPC does not fully reflect the natural diversity in terms of lipid composition^79^. Brady *et al*.^80^ determined the thickness of the SR membrane as 4.1 Å, which is matched by our model membrane (average P-P distance).

By obtaining PMFs for different ion types, major binding sites for both monovalent and divalent cations were identified. The PMFs for cations, shown in Fig. 2a, have similar features and local minima ①, ②, and, for divalent ions, ③. The PMF shapes, depths, and the free energies of binding are very similar for calcium and barium ions, which is supported by the experimental finding that RyR conducts both ions at similar rates^75^. The less affine binding free energy of magnesium ions in our simulation suggests a higher conductance for those ions compared to barium and calcium. This deviates from previous work, where conductances followed the sequence Ba^2+^ > Ca^2+^ > Mg^2+ ^^75^. The disagreement might be due to non-optimal simulation parameters for divalent ions. For monovalent ions, the PMF for K^+^ is significantly shallower that for Na^+^, indicating a better potassium conductance, which is also seen in experiments^74^.

Our findings, summarized in Fig. 2, reveal residues involved in ion permeation, of which some were already identified by mutagenesis studies as vital for the conduction properties of the receptor. Our results offer an atomistic explanation of their functional importance. Starting at the luminal side, acidic luminal loops create an increasingly attractive PMF for monovalent cations, and to an even larger extent, for divalent cations towards the pore entrance. The consequential local increase of cationic concentrations (e.g. visible in the inset of Fig. 4) has been studied previously^26^. Indeed, Mead-Savery *et al*.^81^ showed that the double neutralization ED4832AA on RyR2, which corresponds to ED4902AA on RyR1, has a significant impact on cation selectivity.

Gao *et al*.^71^ and Wang *et al*.^72^ demonstrated the importance of D4899 and E4900 for the conductance and selectivity of the receptor. Neutralizing the charges of the residues by specific mutations led to decreased currents and a loss of calcium selectivity. The charge-conserving mutations D4899E and E4900D did not have such an effect, which supports our observations that the ion interacts mainly with the charged groups of these residues and that ion conduction is indeed governed by strong electrostatic interactions.

In our simulations, the primary constriction site of 3 Å in the SF is formed by G4894. This explains previous mutation studies where an increase in the side chain volume at this point, by G4894A mutation, dramatically reduces both potassium and calcium currents^70,71^. Out of the residues forming the second PMF basin, the charge-neutralizing mutations D4938N, D4945N, and E4952Q were carried out successfully in experiments^73^. In line with our observation of strong electrostatic interactions governing ion permeation in RyR1, D4938N and D4945N showed a decrease in both current and selectivity^73^. E4952Q did not show a significant effect^73^, however it is located farther away from the permeation pathway. Additionally, E4955Q yielded unchanged calcium and potassium currents in experiments^73^, which is also explained by our simulations: although the residue is part of a pore forming helix, its side chain is facing outwards and hence cannot directly interact with permeating ions.

The PMFs, summarized in Fig. 2, reveal that RyR1 has a high affinity for divalent ions, whereas the monovalent cations K^+^ and Na^+^, abundant in the SR and the cytoplasm, face a significantly shallower free energy well. This observation suggests a large conductance for those ions, as seen during the permeation simulations with potassium ions (see Fig. 4). Indeed, monovalent ions were found to be conducted at much higher rates than Ca^2+^ through calcium channels if no calcium was present^71–73,82–87^. Due to the high calcium affinity, the pore was occupied and blocked at basin ① by at least one divalent ion during all unbiased equilibration simulations with both monovalent and divalent ions present in a simulation box. Therefore, the shallow free energy wells for monovalent cations do not necessarily translate to an increased conductance for those ions under physiological conditions (i.e. with both K^+^ and Ca^2+^ ions available in the solution), which is shown best by the reduced potassium current in our permeation simulations when calcium ions are present. Even when accounting for a probable over-estimation of the calcium affinity in our simulations, which will be discussed in the next paragraph, this observation is consistent with the idea that the RyR achieves its calcium selectivity by expressing over-affinity for calcium ions in the same fashion as previously proposed for both RyR^88–90^ and the L-type calcium channel^91^. In this model, a calcium ion binds tightly to the pore and blocks the permeation of monovalent cations, since their coulombic repulsion is too weak to release the calcium ion from its binding site. Only with the aid of the stronger electrostatic interaction (e.g. a second calcium ion), the first ion can be released from the binding site and permeate the channel. This idea is strongly supported by the finding that the charge of D4899 and E4900 is vital for the calcium selectivity^71,72^. The high calcium affinity also suggests a cooperative calcium conduction mechanism, as observed in both Na^+^ and K^+^ channels^66,92,93^ which could be investigated using multi-ion PMFs in future studies. Indeed, we observe multiple ions occupying binding site ② during the applied voltage simulations with potassium ions.

Ion permeation simulations with applied voltage were conducted with potassium, as attempts with calcium were unsuccessful due to the ions getting stuck in the pore. Indeed, overestimated binding affinities of divalent ions, and of calcium ions in particular, are a known problem in classical force fields, including CHARMM36, which we used in this study. In this classical representation, partial charges are fixed during the simulation, which makes the model unable to cover electrostatic polarization effects^94^. Those effects are particularly important for divalent ions due to their strong electrostatic field. It has been shown that the lack of polarization effects leads to an overestimation of ion-protein affinity in the order of 20% for calcium ions^95^.

The conductance of (668 ± 26) pS, as obtained using potassium ions, for which the polarization problem is much less critical^95^, is in very good agreement with the experimental values, ranging between 700 and 800 pS^71–74^, which demonstrates the validity of our simulation approach. More careful analysis of the ion permeation simulations revealed possible additional modes of exiting the RyR into the cytoplasm. Although the observation is made on a reduced structure, visual inspection of the full model as well as of the cryo-EM density by des Georges *et al*.^22^ indicates that the additional pathways are not blocked by the remaining parts of the receptor (see Supplementary Material Fig. 12). It is an intriguing idea to link those additional pathways to the three commonly observed subconducting states at 1/4, 2/4 and 3/4 of the maximal conductance of RyR1^96^ that were observed for different ion species, including potassium^97^. In the past, other groups have explained the subconducting states by four individual contributions of the four protomers^8,97–100^. Although today’s high resolution 3-D reconstructions clearly show a single, central pore, our study suggests that the ionic current could indeed split up, forming multiple conduction pathways.

The presented MD simulations of RyR1, which are based on the latest structural data, provide new insights into the conduction mechanism of the receptor and its energetics. The PMFs, calculated for different ion types, showed that the residues D4899, E4900, G4894, Q4933, I4937, D4938, E4945, E4952, D4966, D5026 and E5033 are the most important for efficient ion permeation, in a broad agreement with available mutagenesis data. The stronger affinity of RyR1 for divalent ions compared to monovalent ions indicates that the receptor achieves calcium selectivity by over-affinity. We observed this effect directly in ion permeation simulations with applied voltage, using potassium ions. The calculated conductance for K^+^ ions is in excellent agreement with experimental estimates. The analysis of the conduction pathways revealed that some ions leave the receptor via previously unknown exits pathways, which we suggest as a possible explanation for the commonly observed subconductance states of RyR1.

## Electronic supplementary material

Supplementary Information

Video 1

Video 2
