# Supplementary material for: In silico assessment of the conduction mechanism of the Ryanodine Receptor 1 reveals previously unknown exit pathways
Source: Sci Rep. 2018 May 2;8:6886. doi: 10.1038/s41598-018-25061-z (PMC5932038; doi:10.1038/s41598-018-25061-z)
Supplement: Supplementary file 1 — Supplementary Information [file 41598_2018_25061_MOESM1_ESM.pdf]

Supplementary Material:

In silico assessment of the conduction mechanism of the ryanodine receptor 1 reveals previously unknown exit pathways

Leonard P. Heinz[1,†,*], **Wojciech Kopec**[2], **Bert L. de Groot**[2], **and Rainer H.A. Fink**[1]

[1]Medical Biophysics Unit, Medical Faculty, Institute of Physiology and Pathophysiology, Heidelberg University, 69120 Heidelberg, Germany

[2]Computational Biomolecular Dynamics Group, Max Planck Institute for Biophysical Chemistry, 37077 Göttingen, Germany

[*]lheinz@gwdg.de

[†]Present address: Department of Theoretical and Computational Biophysics, Max Planck Institute for Biophysical Chemistry, 37077 Göttingen, Germany

Supplementary videos

Video 1. A low-pass filtered trajectory of K^+-permeation under 0.3 V applied voltage. Residues colored according to Fig. 2 of the main text.

Video 2. Low-pass filtered trajectories of potassium ions, representatives for each of the 3 modes of exiting the channel. The simulation was done under 0.3 V applied voltage. Residues colored according to Fig. 2 of the main text.

On the modeling and equilibration

Modeling

Table 1 provides a list of loop segments that were unresolved in all of the referenced cryo-EM datasets and thus needed to be fitted using the Modeller software[1–4]. All loop models went through multiple successions of conjugate gradient optimization and simulated annealing. The final model was chosen among 100 independent runs based on the overall score.

Table 1. Number of loop segments modelled with the Modeller software and their respective sizes. E.g. 11 loop segments with lengths between 1 and 5 residues were modeled in total. Of those, 2 are part of the reduced model and none is located in the pore region.

**Number of residues**	**Number of loops**		
	full model	reduced model	pore region
1–5	11	2	0
6–10	7	0	0
12	2	0	0
18	1	0	0
**total**	21	2	0

In addition to loop segments, homology models for two larger unresolved parts were constructed externally. Those segments are given by H1296–N1418 and Q3570–L3644.

The templates used for the full model are shown graphically in Fig. 1.

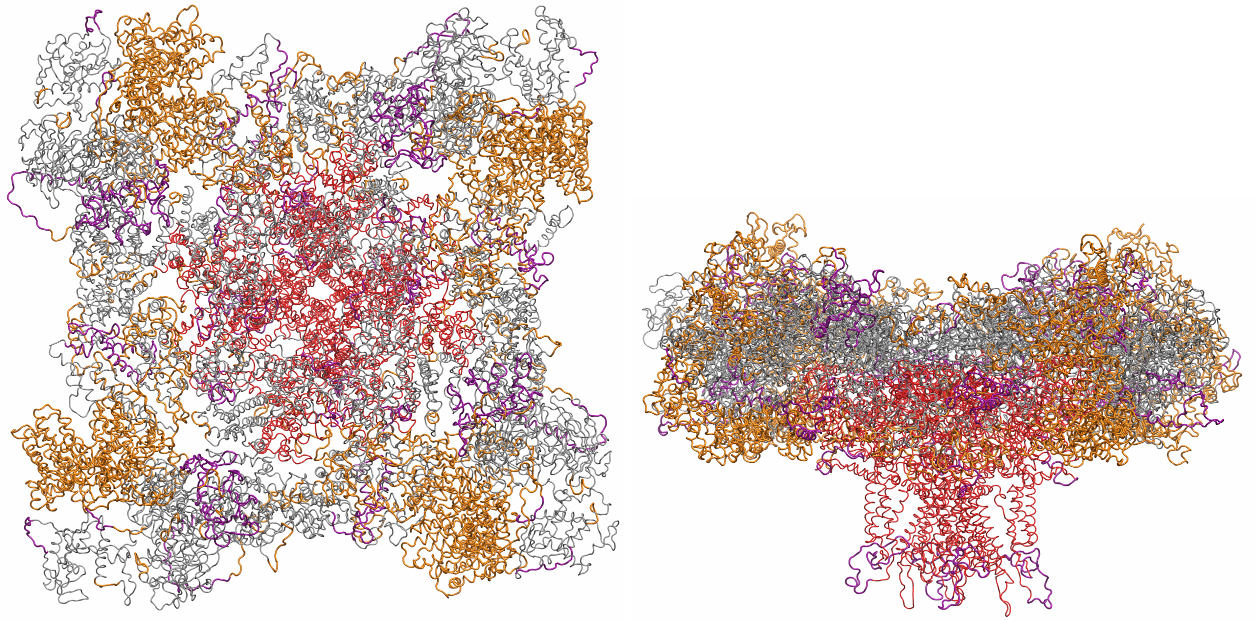

Figure 1. Templates used for each section of the model: red: Yan et at[5] (pore region), gray: Yan et al[5] ('crown'), orange: Zalk et al[6], purple: loops/homology models.

Equilibration of closed-state RyR1

Fig. 2 shows the RMSD during 100 ns of equilibration of the full receptor in its closed state. As expected, 100 ns is not sufficient to fully equilibrate the entire receptor, consisting of almost 20000 amino acids. It is, however, enough to equilibrate the subsystem that would make up the reduced model, including the important pore region.

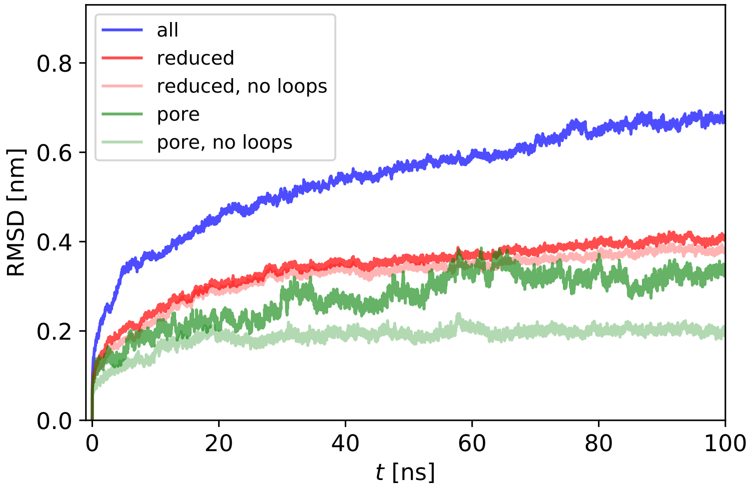

Figure 2. RMSD value based on Cα atoms over the course of 100 ns. The values are based on all residues (blue), the residues that would later make up the reduced system (red) and the pore region (green). The semi-transparent lines show the RMSD of the respective system, excluding the extremely flexible luminal loops.

Equilibration of open-state RyR1

Fig.s 3 and 4 provide information about the equilibration period of the reduced model after it was steered in the open conformation. Here, open-state restraints were kept on every 4th Cα atom for the first 30 ns.

To quantify whether a reclosing movement occurred during the equilibration, as well as to provide a further measure of the similarity between the PDB template and our model, we calculated the projection of the equilibration trajectories on the difference vector between the closed model and the open cryo-EM structure in configuration space.

Let x_{closed} and x_{open} be the configuration space vectors describing the closed state model and the open cryo EM structure respectively. We define the projection λ as

$$\lambda(t) = \frac{(x(t) - x_{\text{closed}}) \cdot (x_{\text{open}} - x_{\text{closed}})}{\|x_{\text{open}} - x_{\text{closed}})\|_2^2},$$

where $x(t)$ corresponds to the configuration at time t of the trajectory. According to the definition above, $\lambda = 0$ corresponds to a fully closed structure and $\lambda = 1$ indicates a fully open conformation. Fig. 3 shows $\lambda(t)$ for both, the equilibration runs with and

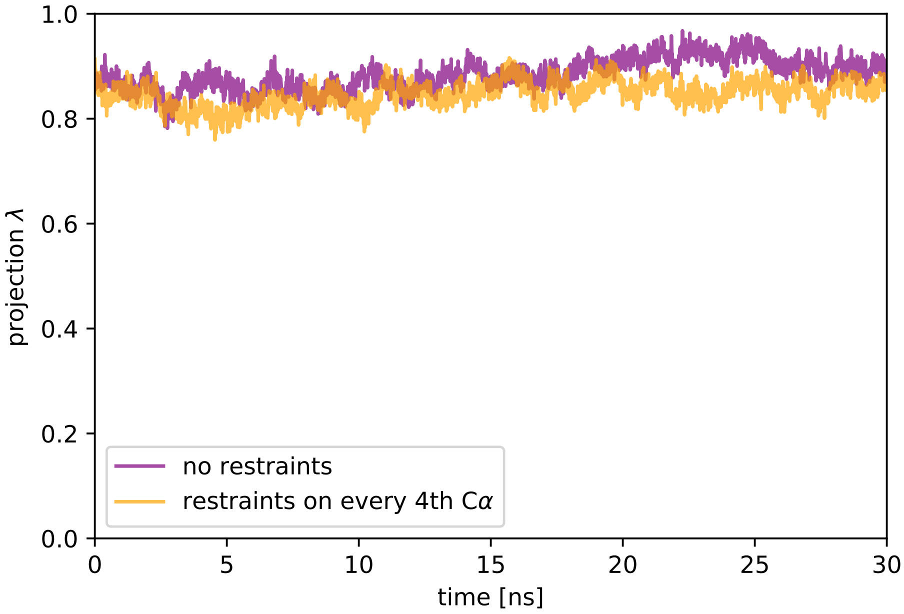

Figure 3. Normalized projection of the equilibration simulations on the difference vector between the closed model and the open cryo EM structure in configuration space. Values of 0 and 1 correspond to fully closed and fully open structures respectively.

without open-state restraints on every 4th Cα atoms. The trajectories fluctuate around $\lambda = 0.85$ which denotes configurations very close to the open cryo-EM structure. In particular, there is no drift towards smaller values of λ, which would denote a reclosing movement. Furthermore, there is no difference between the trajectories with and without restraints, which justifies the lack of open-state restraints during umbrella sampling.

Fig. 4 shows the RMSD during the equilibration period of the reduced model after it was steered in the open conformation. For the first 30 ns of the simulation run, open-state restraints were kept on every 4th Cα atom. Later the restraints were released, causing an RMSD increase of ≈ 0.1 nm. The umbrella sampling simulations as well as the applied voltage simulations were started from the structure at 60 ns and the equilibration was continued to a total of 200 ns to assess its convergence. The pore RMSD leveled off at ≈ 0.35 nm.

Note that the RMSD does not exactly start at 0, because after the steered simulation run, the model is very similar but not perfectly identical to its template due to thermal fluctuations.

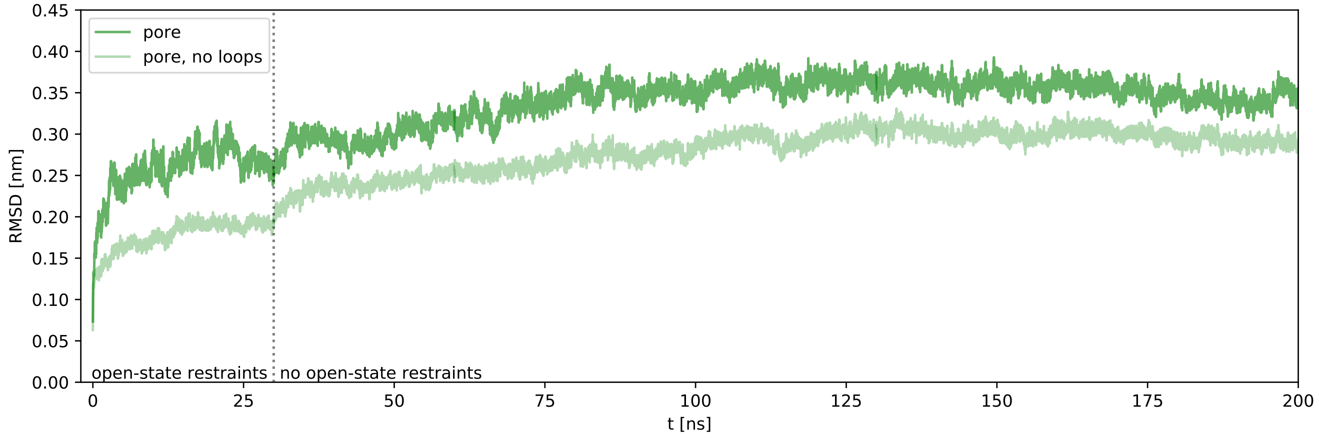

Figure 4. RMSD values with respect to the open-state template (PDB: 5TAL) of the pore-region during the equilibration of the open-state model. As indicated by the dashed line, open-state restraints on every 4th Cα atom were used during the first 30 ns, after which they were released.

Fig.s 5 and 6 show overlays of our equilibrated model, as used for the applied voltage simulations with restraints on every 4th Cα atom, with the PDB template and the cryo-EM density respectively. We note that the structures are generally in good agreement, as also indicated by the RMSD of 2.5 Å.

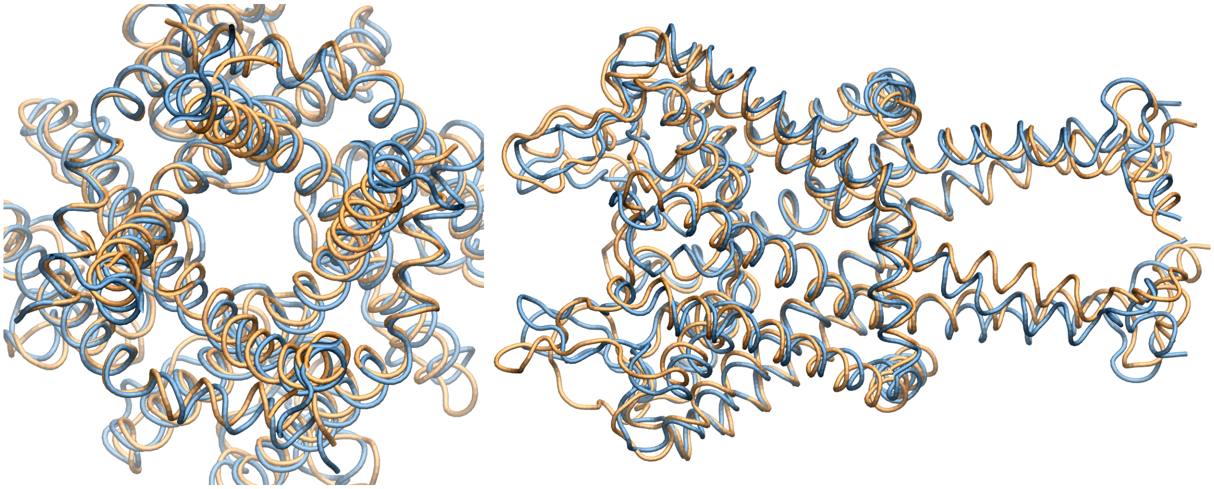

Figure 5. Overlay of the pore regions of the open-state pdb-template (blue) and the model after equilibration (orange). The images show a top view along the pore axis and a side view respectively.

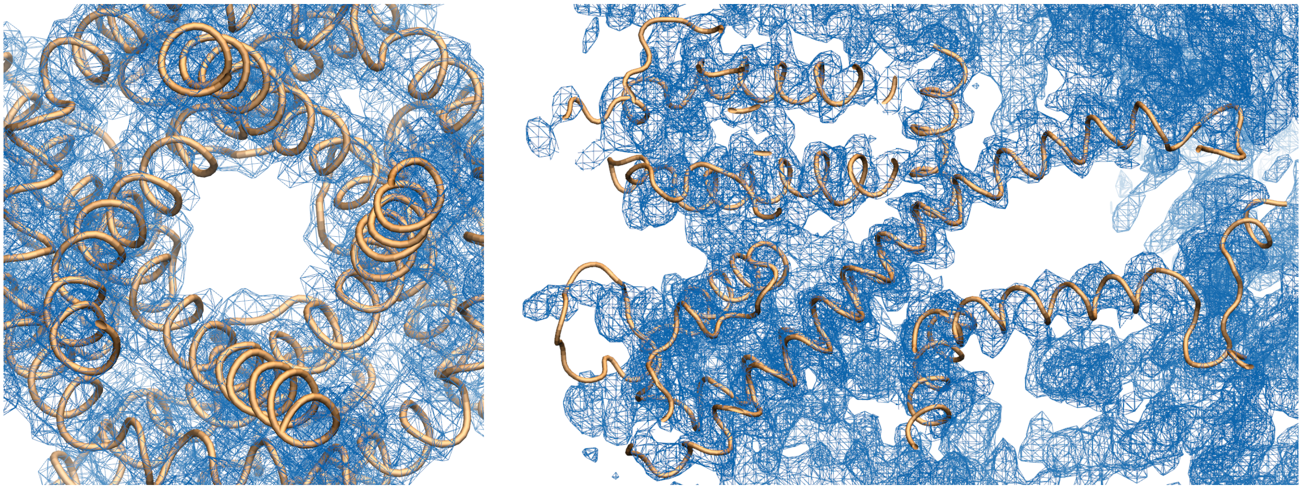

Figure 6. Overlay of the pore regions of the open-state cryo-EM density (blue) and the model after equilibration (orange). The images show a top view along the pore axis and a side view respectively.

On the Gromacs implementation of the energy-step method

The energy-step, used to restrain ions in their respective compartment, was implemented into Gromacs by expanding upon the set of existing flat-bottom potentials. Fig. 7 shows an example of a energy step. The potential is flat in front and behind a transition region - i.e. there will be no force acting on the particles subject to the potential. A constant force f is exerted in the transition region with width d. The height of the step is given by $d \cdot f$.

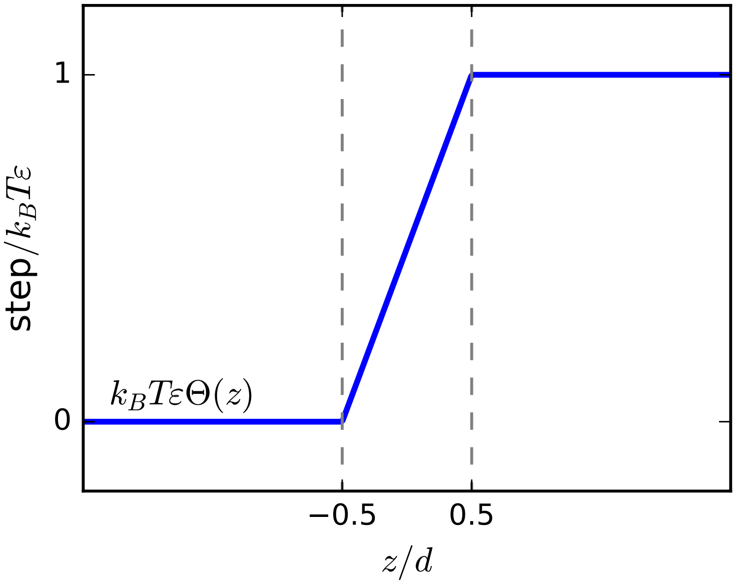

Figure 7. Energy step of width d and height $d \cdot f = k_BT\varepsilon$. In our current implementation, the step width d is hard coded as 0.5 nm. However, treatment of d as a further parameter is possible in future updates.

Just as any other position restraint, energy-steps can be added to a moleculetype in the topology file. In contrast to the existing implementation of flat-bottomed potentials, the reference points are not determined by the initial particle positions, but the position of the step can be chosen freely as a parameter. Our implementation allows for energy steps in x,y and z directions. Multiple steps can be combined; for instance six steps could be used to restrain atoms in a cuboid-shaped volume.

Adding the lines below to a moleculetype-section in the topology file restraints the movement of the first atom (`i=1`) in z-direction (`g=11`).

```
[ position_restraints ]
;  i  funct     g       z0       f
   1     2     11     0.25   10000
```

The step is located at `z0=0.25` nm and the force in the transition region is provided by `f=10000` kJ/mol/nm. Steps facing in x and y-direction are parameterized by `g=9` and `g=10` respectively. Smaller values of the `g` parameter lead to the stock-Gromacs flat-bottom potentials, as described in the section 'Flat-bottomed position restraints' of the Gromacs manual.

Our implementation of the energy-step is available on request.

On the convergence of umbrella sampling

We address the quality of convergence of the umbrella sampling simulations on the example of Ca^{2+}. The histogram, provided in figure 8, shows that the entire interval along the reaction coordinate is well sampled and that neighboring windows strongly overlap.

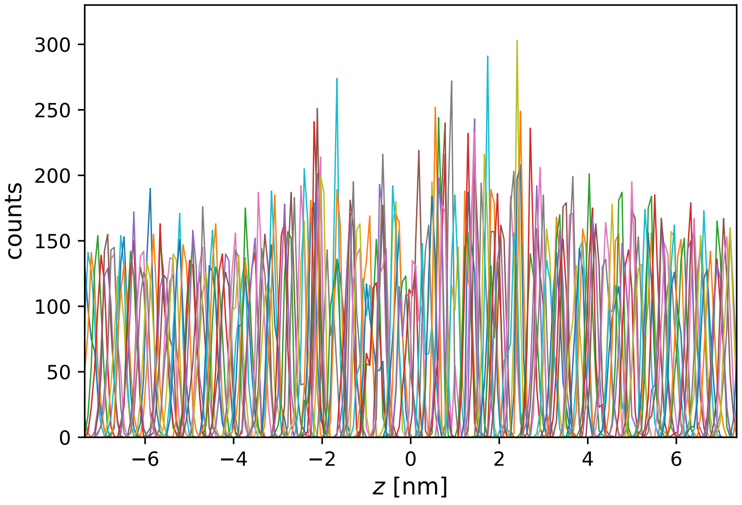

Figure 8. Histograms of all umbrella sampling windows of the calcium ion.

To assess the convergence in a more quantitative fashion, we calculated the potential of mean force while every 2nd, 3rd and 4th sampled windows were removed from analysis respectively. The results are summarized in Fig. 9, where the PMF based on the full set of windows is shown as well. The maximal deviation from the full PMF amounts to 6.6 kJ/mol. This is in agreement with the 1σ error range of ± 5.4 kJ/mol as obtained by bootstrapping. Furthermore we shorten the analyzed length of each umbrella window by up to 400 ps and compare it to the full data set in Fig. 10.

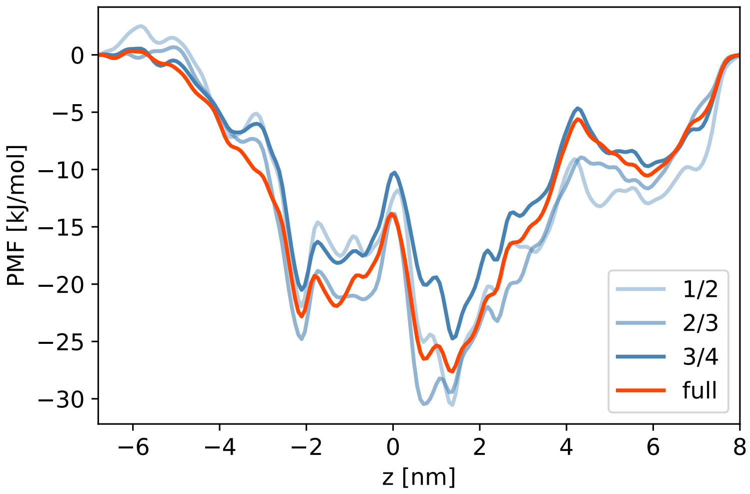

Figure 9. PMF of a calcium. The PMF based on all umbrella windows is shown in orange. The blue curves show PMFs based on only 1/2, 2/3 and 3/4 of all windows.

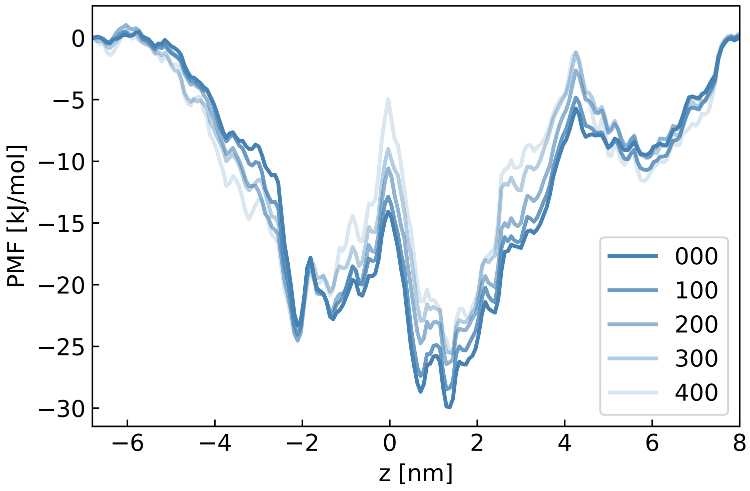

Figure 10. PMF of a calcium, based on the full trajectory of each window (000) as well as with the first 100, 200, 300 and 400 ps excluded from analysis.

To further address the question whether 0.6 ns for each of the 209 umbrella windows are sufficient to converge the PMF as well as relax the channel properly, we increased sampling for Ca^{2+} ions and observed the corresponding changes to the PMF. 10 umbrella windows, located in the core of the channel, were substituted by longer simulation runs, which lasted 3 ns. Comparison between the old PMF and the new PMF with increased sampling, shown in Fig. 11, shows no significant change.

Calculating hydration numbers

We calculated hydration numbers for of both first and second hydration shells for all ion types along the pore axis. Hydration numbers were determined by counting the number of water molecules around the ion closer than cut off radii, corresponding the first two hydration shells, in the final frames of each of the umbrella sampling windows. Local averages along the pore axis were taken using a Gaussian filter with a standard deviation of six windows. The cut off radii were determined based the radial distribution functions (RDFs) of a single ion in bulk water, which was simulated for 10 ns. The first and second local RDF

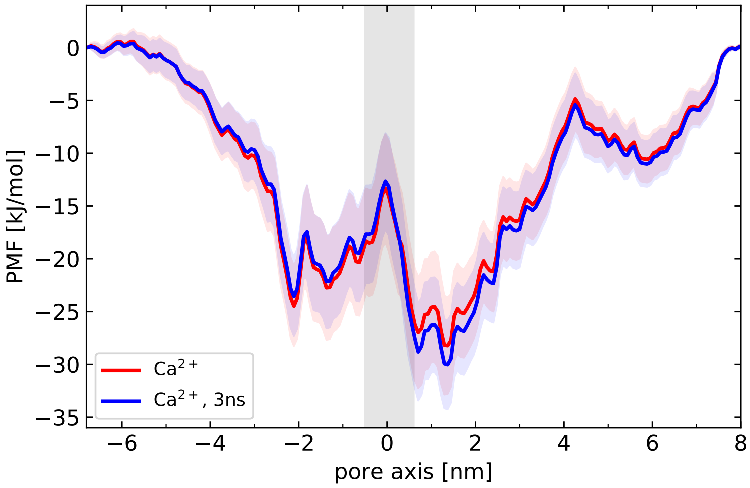

Figure 11. Comparison between the old PMF (red) and the new PMF with increased sampling (blue) for Ca^{2+} ions. For the latter, the 10 windows, located in the shaded interval, were rerun for 3 ns.

minima yielded the hydration shell radii as summarized in table 2.

Table 2. Position of the first and second RDF minima in water, as determined in simulations of 10 ns in bulk phase.

Ion	1st minimum	2nd minimum
	[nm]	
Ba^{2+}	0.35	0.60
Ca^{2+}	0.30	0.55
Mg^{2+}	0.25	0.50
K^{+}	0.35	0.60
Na^{+}	0.32	0.57
Cl^{-}	0.38	0.63

On the classification of the ion permeation trajectories

A heuristic approach was adopted, in order to distinguish and to quantify the different observed modes of exiting the channel, such as visualized in Fig. 5 of the main text.

Classification was done using a scoring function

$$S_{ij} = \sum_{k=1}^{N_f} \exp\left(-\frac{\|\vec{x}_i^{(k)} - \vec{r}_j\|^2}{d^2}\right), \quad (1)$$

which scores the likelihood that ion i passed through exit j during the course of its trajectory $\{\vec{x}_i^{(k)} | k \in \{1, \ldots, N_f\}\}$. Here, N_f is the number of captured frames and $\vec{r}_j$ is the exit point of pathway j. The parameter d determines up to which distance to an exit point a trajectory still contributes significantly to the score. Reasonable classification was obtained by choosing $d = 1$ nm. Trajectory i was considered unclassifiable if the second largest entry in $\{S_{i1}, S_{i2}, S_{i3}\}$ was greater than 80% of the largest entry. In the other case, the ion was classified as having passed through the highest scoring exit.

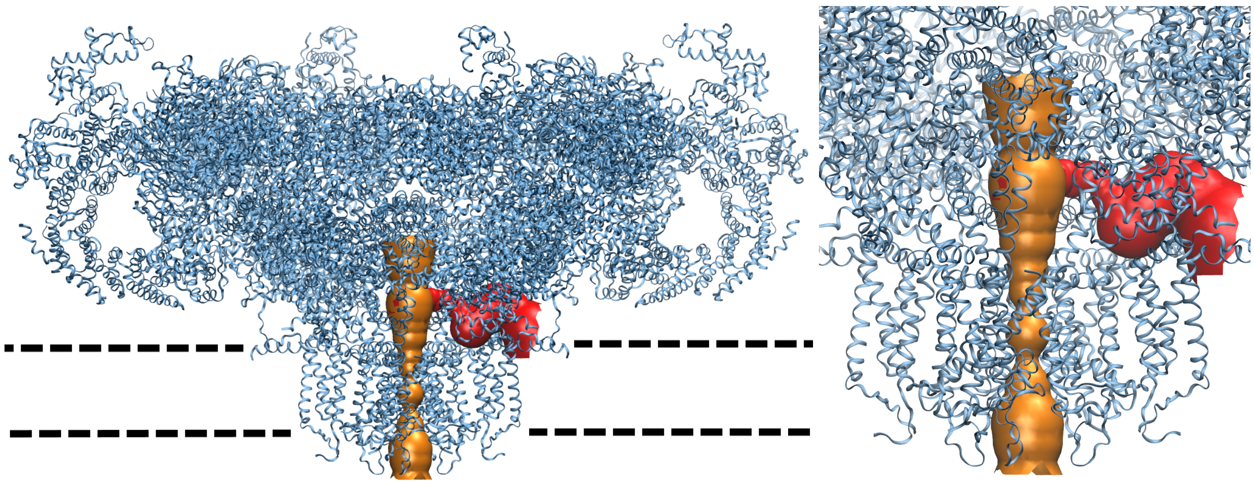

Figure 12. Visualizations[7] of the main exit pathway (orange) as well as one of the lateral pathways (red) based on the full PDB template (5TAL). The position of the membrane is marked indicated by the black dashed lines.

References

1. Sali, A. & Blundell, T. Comparative protein modelling by satisfaction of spatial restraints. *Protein structure by distance analysis* **64**, C86 (1993).

2. Marti-Renom, M. A. *et al.* Comparative protein structure modeling of genes and genomes. *Annu. review biophysics biomolecular structure* **29**, 291–325 (2000).

3. Fiser, A., Do, R. K. G. & Šali, A. Modeling of loops in protein structures. *Protein science* **9**, 1753–1773 (2000).

4. Webb, B. & Sali, A. Comparative protein structure modeling using modeller. *Curr. protocols bioinformatics* 5–6 (2014).

5. Yan, Z. *et al.* Structure of the rabbit ryanodine receptor RyR1 at near-atomic resolution. *Nat.* **517**, 50–55 (2015).

6. Zalk, R. *et al.* Structure of a mammalian ryanodine receptor. *Nat.* **517**, 44–49 (2015).

7. Smart, O. S., Neduvelil, J. G., Wang, X., Wallace, B. & Sansom, M. S. HOLE: a program for the analysis of the pore dimensions of ion channel structural models. *J. molecular graphics* **14**, 354–360 (1996).
